# A social network perspective on social cues for COVID risk perception

**DOI:** 10.1038/s41598-025-99673-7

**Published:** 2025-04-29

**Authors:** Helge Giese, F. Marijn Stok, Wolfgang Gaissmaier, Odette Wegwarth

**Affiliations:** 1 Heisenberg Chair for Medical Risk Literacy and Evidence-based Decisions, Charité – Universitätsmedizin Berlin, Charitéplatz 1, 10117 Berlin, Germany; 2 Department of Psychology, University of Konstanz, Konstanz, Germany; 3 Department of Interdisciplinary Social Science, Utrecht University, Utrecht, The Netherlands; 4 National Institute of Public Health and the Environment, Bilthoven, The Netherlands; 5 Center for Adaptive Rationality, Max Planck Institute for Human Development, Berlin, Germany

**Keywords:** Social networks, Optimistic bias, Social judgement, Social amplification of risk, Availability by recall, Psychology, Public health

## Abstract

Lay perceptions of risks are often at odds with their empirical assessments, particularly regarding risks of peers. Going beyond only considering perceived peer risks, this study explored whether the actual social environment of a person informs their individual COVID risk perception. A cohort of Psychology freshmen (*N* = 88; academic year 2021/22) was surveyed about their COVID infection status in the past year, prevalence estimates within their cohort, and their social relationships. They were further queried on their expected susceptibility for a COVID infection within the forthcoming month and the potential severity of such an infection. Average student estimation of 1-year prevalence rates (71.8%) were in line with the self-reported prevalence of the cohort (69.8%, *p* = .110) and took infection prevalence in their social circle into account (β = 0.24, *p* = .025). This social circle prevalence also contributed to the individual assessment of COVID susceptibility (β = 0.24, *p* = .031), but not the severity of the disease (β = 0.05, *p* = .671). These results indicate that the perception of prevalence among peers is not necessarily biased and that social cues of prevalence in the environment are considered when estimating individual susceptibility.

## Introduction

The perception of being at risk is a widely considered motivational factor in health behaviors^1–3^. However, being overly cautious may also have negative consequences such as social isolation^4,5^. Therefore, having a good sense of the likelihood of events like infections is one essential aspect of informed health decision making^6,7^. But how do these perceptions emerge? In this study, we want to scrutinize to what extent this risk perception is informed by cues in one’s social environment in the context of the COVID pandemic.

It has long been recognized that particularly unknown and dread-inducing risks are likely to elevate risk perceptions^8^, but the extent to which a risk is dreaded and affecting large numbers of people at a time must be inferred from external sources. One potential source for this type of information is one’s direct social environment^9,10^: The answer to the question “How many peers did I observe to fall sick?” should help to gauge how susceptible a person is to a disease and be indicative of a certain prevalence. The hypothesis that social cues are used for risk perception, oftentimes referred to as the social circle heuristic^9,10^, is also reinforced by medium associations of perceived social exposure to the COVID virus and COVID risk perceptions^5,11,12^.

However, the social circle heuristic for risk perception has not yet been validated with actual data from the individual social environment, widely assuming that individual perception of one’s social circle is generally accurate. Yet, the positive correlation of risk perception with how people perceive a prevalence in their social surroundings^5,9^ may also be explained by the projection of one’s own risk experience onto the environment^13,14^, thereby exaggerating the assumed role of the social information for risk evaluation. Furthermore, given that a large set of studies indicates that social perception of risk is optimistically biased towards believing oneself to be less at risk compared to peers^15,16^, it is unclear whether the estimation of peer risks, the social environment, or both are systematically biased to overrepresent risk in peers.

### The current study

Therefore, the current study explores (1) how the actual COVID prevalence in one’s social environment and not the mere perception of this prevalence by an individual may shape the perception of COVID risk. To this end, we employ a novel social circle approach, assessing a social peer network and self-indicated 1-year COVID prevalence (the social circle prevalence) as an indicator of each individual’s social environment. In a mediation model, we then test how the COVID prevalence in each individual’s social circle predicts the perception of the entire group’s cohort prevalence and how both the social circle prevalence and the cohort prevalence estimations relate to individual risk perceptions. In addition, the assessment of prevalence in the social circle and in a confined group setting further allows us to explore (2) how COVID prevalence is represented in the social environment (i.e., in the cohort and social circle) and whether prevalence estimations of the surveyed cohort are biased.

## Results

### Social effects on risk perceptions

**Fig. 1:**
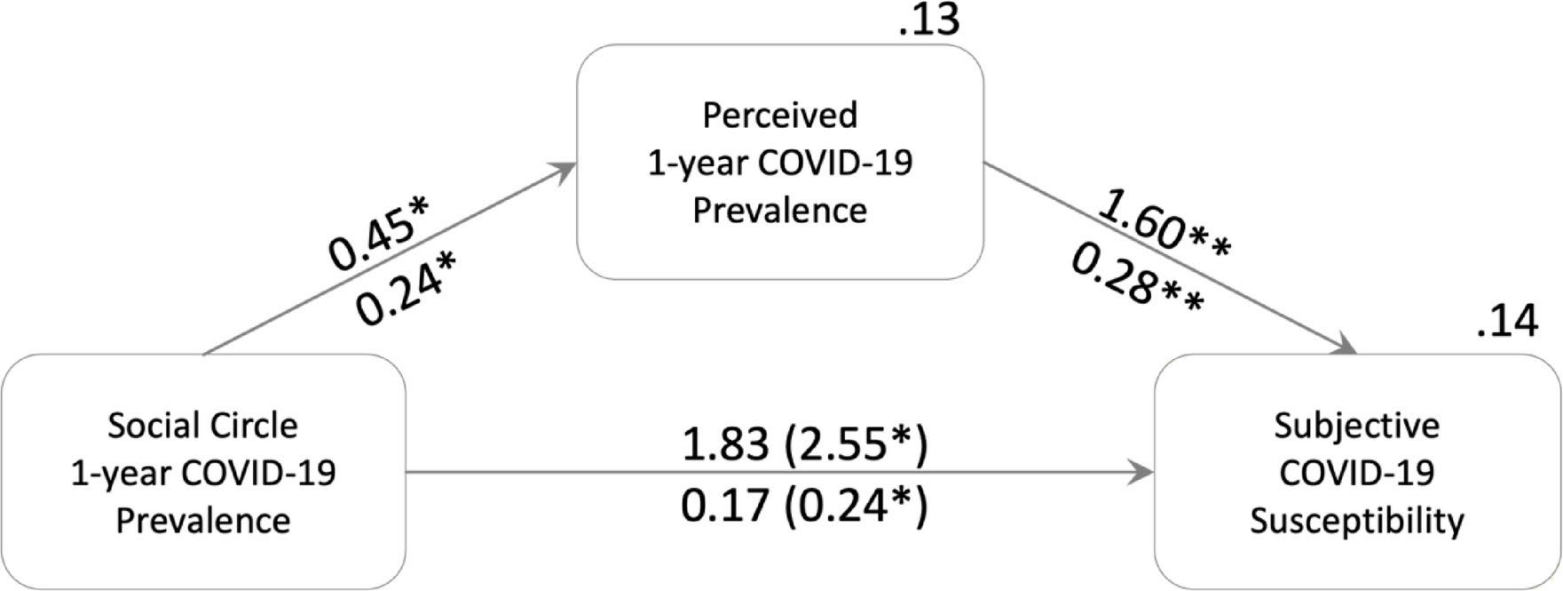
Social Effects on Susceptibility Risk Perception. Unstandardized effects are above, standardized effects below arrows. Total effects are in parenthesis. The effects are controlled for total number of acquaintances in social circle and own infection status. The unstandardized, indirect effect with bias-corrected bootstrap is 0.72 [0.05; 1.96], standardized it is 0.07 [0.01; 0.19]. *R*^2^ are displayed above the boxes of the outcomes, including controls of own infection status and social circle size. *p < .05; **p < .01.

The prevalence in an individual’s social circle affected both estimated cohort prevalence (*b* = 0.45 [0.04; 0.84], β = 0.24 [0.03; 0.45], *p* = .019) and susceptibility (*b* = 2.55 [0.15; 4.45], β = 0.24 [0.03; 0.43], *p* = .025) with a significant indirect effect on susceptibility via estimated cohort prevalence (*b* = 0.72 [0.05; 1.96], β = 0.07 [0.01; 0.19]; Fig. 1, difference between total and direct effect). That means, for each percentage point increase in prevalence within a social circle, the estimated cohort prevalence increased by about 0.5% points, and having only infected peers in one’s circle increased subjective susceptibility by half of the 5-point scale compared to having no cases in one’s circle. Neither individual infection status nor social circle size were significantly related to estimated prevalence (status: *b* = − 0.06 [–0.14; 0.02], β_y_ = − 0.32 [–0.77; 0.12], *p* = .150; size: *b* = 0.00 [–0.00; 0.01], β = 0.22 [–0.00; 0.42], *p* = .064) and subjective susceptibility (status: *b* = 0.16 [–0.26; 0.58], β_y_ = 0.16 [–0.26; 0.58], *p* = .483; size: *b* = 0.00 [–0.01; 0.01], β = 0.01 [–0.19; 0.20], *p* = .932). Likewise, the severity estimation of COVID was unrelated to individual infection (*b* = − 0.11 [–0.63; 0.40], β_y_ = − 0.12 [–0.64; 0.41], *p* = .629), social circle size (*b* = − 0.00 [–0.02; 0.02], β = − 0.00 [–0.30; 0.27], *p* = 1.00), prevalence in the social circle (*b* = 0.54 [–1.59; 2.95], β = 0.05 [–0.16; 0.29], *p* = .646), and cohort prevalence estimation (*b* = 0.27 [–1.07; 1.54], β = 0.05 [–0.20; 0.28], *p* = .671).

### Biases in estimations of prevalence

**Fig. 2:**
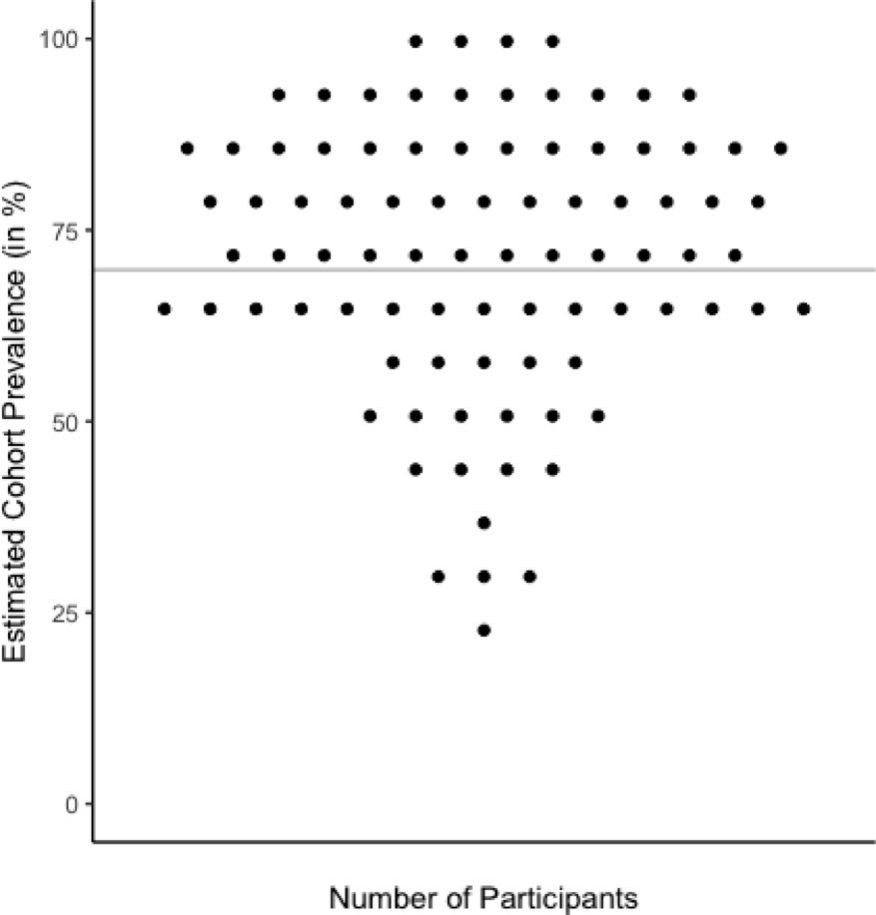
Individual Estimated 1-year COVID-19 Cohort Prevalence Compared to the Overall Cohort Prevalence. The line represents the cohort prevalence. Each point represents an individual estimate. The absolute estimation error of an individual can be inferred from the deviation of the individual estimate from the line.

Overall, the estimation of COVID prevalence within the cohort was not detectably biased compared to all self-reported infections (*V* = 2343, *p* = .110): Students estimated on average that 71.8% [68.0%; 75.5%] (SD = 17.6%) of the cohort experienced a COVID infection during their first year of study and 69.8% (60 out of 86) of the cohort indeed self-reported an infection. The average reported prevalence within individual social circles at 66.9% [64.8%; 68.9%] (SD = 9.5%) was overall slightly lower than the cohort estimate (*V* = 2589, *p* = .004, *M*_Δ_ = 4.9% [1.2%; 8.6%]) and the cohort prevalence (*V* = 1333, *p* = .009). The average estimation error, i.e., the mean absolute deviation of the student estimate from the cohort prevalence, was 14.3 [12.4; 17.0] percentage points (SD = 10.3) and significantly higher than 0 (*p* < .001) showing that —while overall unbiased — individual estimates typically deviated from the cohort prevalence (Fig. 2).

## Discussion

The external social environment of the individual correlated with both perceived cohort prevalence and individual susceptibility, corroborative of the notion that social circle prevalence could be a cue used to infer personal risk^9,13^. In contrast, the severity estimation was not affected by any of the prevalence assessments, indicating that the students were able to accurately differentiate between the two risk measures. Still, there was a considerable amount of dispersion in the estimation of both prevalence and susceptibility **—** other factors not surveyed here must play a considerable role in the estimation process, and the social cues were not perfectly perceived. For instance, a COVID infection and high prevalence might both decrease the prospective risk of contracting the virus via perceived (herd) immunity, but also may elevate it due to exposure^5,12,13^. In addition, because the prevalence assessment and estimation were confined to a specific peer group in the individual’s vicinity, other groups and sources of information could also alter susceptibility perceptions^5,18^. Similarly, we have not assessed the individual exposure to the social circle, which is why we cannot determine individual differences in the role of social circle prevalence as a cue for risk evaluation.

The estimated prevalence closely matched the prevalence in the cohort and was not overestimated, exhibiting no apparent bias regarding exaggerating peers’ COVID risk. Furthermore, there was no indication of overrepresentation of COVID risk in peers: COVID-sick people were rather less part of social circles compared to the people who stayed healthy, which may be seen as a sign of social distancing during periods of COVID^4^. Studies that show that COVID susceptibility of peers is systematically rated higher than personal susceptibility^16^ may thus not be explained by an overrepresentation of events in the social environment, but rather may reflect a misrepresentation of one’s own risks^17^.

This study has some limitations. First, all the analyses are based on self-reports. Therefore, over- or underreporting of COVID may be introduced by the social desirability of responses and selective dropouts. Similarly, biases in peer estimation could still be found for low base rate risks, as larger biases are typically observed in these contexts^14,19^. Therefore, generalization of all results to larger, more general populations with a non-biased gender distribution still needs to be achieved, and additional experimental designs, possibly similar to the approach in Giese et al.^20^, are warranted for causal attribution, as all findings reported in this study were correlational and explorative. This is particularly true as non-significant differences might alternatively be explained by a lack of power. Therefore, some caution in interpreting the results is advised until these have been replicated.

In summary, we used a novel network-based technique to assess how the social environment accounts for risk perception. With this technique, we found that COVID prevalence estimation is not detectably biased and takes social information into account. Future studies may use the technique to further test how the perception of biases arises, also because the approach allows for defining biased risk perception on an individual level.

## Methods

### Participants and procedure

All 116 first-year Psychology students in 2021 at the University of Konstanz were invited to participate in a longitudinal social network study during their first week at university. One hundred (86 females; *M*_*Age*_ = 20.6, *SD* = 3.5) of them agreed to participate, signing an informed consent sheet in November 2021. The data presented here is a cross-sectional selection of a larger project with a longitudinal assessment of more traits, behaviors, and cognitions in a social network^20^. The data was assessed in a series of online surveys with links personalized to the consenting participants for seven measurement points. For completion of the full longitudinal study, participants received up to 3.5 h of course credits plus a maximum of 23.50 €, dependent on how many time-points were successfully completed within a 1-week deadline. The data concerning the current research question was obtained at the beginning of November 2022, with 88 of the students participating. The drop-out was selective neither with respect to gender (*p*_*Fisher−exact*_ = 0.103) nor age (*W* = 384.5, *p* = .505). During the academic year 2021/22, the students attended both online and offline seminars and lectures together. The study was approved by the University of Konstanz IRB (32/2021) and was carried out in accordance with the Declaration of Helsinki. The sample size was sufficient to detect medium sized effects of *r* = .29 with a power of 0.8 for α ≤ 0.05. The tests in this study were not pre-registered and should be considered exploratory.

### Measures

#### COVID infection status and cohort prevalence

Participants indicated whether they contracted COVID within the last year (“*Have you been infected with COVID within the last year?*”). They had the option not to disclose their infection status. The proportion of infected participants is considered the *Cohort Prevalence* in COVID infections.

#### Risk perception

We assessed individual risk perception of COVID with both a measure of subjective susceptibility (“*How likely do you think it is*,* that you will get infected with COVID within the next month*?” *very unlikely* – *very likely*, *M =* 2.96, *SD* = 1.00) and infection severity (“*How severe are the following events*,* in case they occur to you personally? Corona infection*” *not at all severe* – *very severe*, *M =* 2.76, *SD* = 0.97) on 5-point scales.

#### Prevalence estimation

Participants were asked about the proportion of people in their cohort having had COVID within the past year at least once (“*How high do you estimate the proportion of people in your class that have been infected with COVID at least once within the past year?*” *0–100*).

#### Social circle prevalence

Each individual rated their relationship with all other participating students by marking on a list of names which persons they were acquainted with as an indication of their social circle (*M =* 28.2, *SD* = 14.4). The marking instructions specified that the participants should consider a person an acquaintance if they knew that person better than sight/talked to this person within the past half-year period at least once for a longer time. For individual predictions, the fraction of infected people within the social circle was inferred from the self-reported status of all individual acquaintances as social circle prevalence in % ranging from 1 to 100 (see^14,20^).

### Analyses

To estimate how the social circle affected risk perception, we used a path model (see Fig. 1) controlling all outcomes for dummy-coded individual infection status (no as “1”) and social circle size (number of acquaintances indicated). For these path models, we used the lavaan 0.6–16 package with ML estimation to test the effects of the social circle on risk perception. For indirect mediation effect estimation, we used bias-corrected 10,000 sample bootstrapped confidence intervals. Participants not disclosing their infection status (*n* = 2) were treated as missing values for path models and ignored for computing COVID prevalence.

The estimated COVID cohort prevalence and the social circle prevalence were compared with the actual self-reported cohort prevalence in one-sample Wilcoxon tests. To account for potential oversampling of particularly well-known people in one’s social circle, the perceived prevalence was also compared to social circle prevalence reported by the social circle of each individual in a paired Wilcoxon test. A one-sample Wilcoxon test was additionally performed to compare the estimation error against 0. Confidence intervals for these statistics were inferred from respective t-tests.

## Data Availability

The datasets generated and/or analysed during the current study are not publicly available to protect the rights of the participants and avoid identifiability but are available from the corresponding author on reasonable request.
